# Epidemiology and Clinical Characteristics of Respiratory Infections Due to Adenovirus in Children Living in Milan, Italy, during 2013 and 2014

**DOI:** 10.1371/journal.pone.0152375

**Published:** 2016-04-05

**Authors:** Susanna Esposito, Alberto Zampiero, Sonia Bianchini, Alessandro Mori, Alessia Scala, Claudia Tagliabue, Calogero Sathya Sciarrabba, Emilio Fossali, Antonio Piralla, Nicola Principi

**Affiliations:** 1 Pediatric Highly Intensive Care Unit, Department of Pathophysiology and Transplantation, Università degli Studi di Milano, Fondazione IRCCS Ca’ Granda Ospedale Maggiore Policlinico, Milan, Italy; 2 Emergency Unit, Fondazione IRCCS Ca’ Granda Ospedale Maggiore Policlinico, Milan, Italy; 3 Molecular Virology Unit, Microbiology and Virology Department, Fondazione IRCCS Policlinico San Matteo, Pavia, Italy; University of Hong Kong, HONG KONG

## Abstract

To evaluate the predominant human adenovirus (HAdV) species and types associated with pediatric respiratory infections, nasopharyngeal swabs were collected from otherwise healthy children attending an emergency room in Milan, Italy, due to a respiratory tract infection from January 1 to February 28 of two subsequent years, 2013 and 2014. The HAdVs were detected using a respiratory virus panel fast assay (xTAG RVP FAST v2) and with a HAdV-specific real-time polymerase chain reaction; their nucleotides were sequenced, and they were tested for positive selection. Among 307 nasopharyngeal samples, 61 (19.9%) tested positive for HAdV. HAdV was the only virus detected in 31/61 (50.8%) cases, whereas it was found in association with one other virus in 25 (41.0%) cases and with two or more viruses in 5 (8.2%) cases. Human Enterovirus/human rhinovirus and respiratory syncytial virus were the most common co-infecting viral agents and were found in 12 (19.7%) and 7 (11.5%) samples, respectively. Overall, the HAdV strain sequences analyzed were highly conserved. In comparison to HAdV-negative children, those infected with HAdV had a reduced frequency of lower respiratory tract involvement (36.1% vs 55.2%; p = 0.007), wheezing (0.0% vs 12.5%; p = 0.004), and hospitalization (27.9% vs 56.1%; p<0.001). Antibiotic therapy and white blood cell counts were more frequently prescribed (91.9% vs 57.1%; p = 0.04) and higher (17,244 ± 7,737 vs 9,565 ± 3,211 cells/μL; p = 0.04), respectively, in children infected by HAdV-C than among those infected by HAdV-B. On the contrary, those infected by HAdV-B had more frequently lower respiratory tract involvement (57.1% vs 29.7%) but difference did not reach statistical significant (p = 0.21). Children with high viral load were absent from child care attendance for a longer period of time (14.5 ± 7.5 vs 5.5 ± 3.2 days; p = 0.002) and had higher C reactive protein levels (41.3 ± 78.5 vs 5.4 ± 9.6 μg/dL; p = 0.03). This study has shown that HAdV infections are diagnosed more commonly than usually thought and that HAdVs are stable infectious agents that do not frequently cause severe diseases. A trend toward more complex disease in cases due to HAdV species C and in those with higher viral load was demonstrated. However, further studies are needed to clarify factors contributing to disease severity to understand how to develop adequate preventive and therapeutic measures.

## Introduction

Human adenoviruses (HAdVs) are a group of at least 68 non-enveloped viruses containing double-stranded linear DNA [1]. They belong to the family *Adenoviridae*, genus *Mastadenovirus* and are categorized into seven species (A-G) according to their biophysical, biochemical, and genetic characteristics. Moreover, in each of these species, several types have been identified [1]. Species identity strongly correlates with antigenicity, epidemiologic characteristics, clinical manifestations of HAdV infection, and *in vitro* response to some antiviral drugs [2, 3]. From a clinical point of view, species D (HAdV-D8, HAdV-D19, and HAdV-D37) is usually associated with the development of conjunctivitis; species F (HAdV-F40 and HAdV-F41) is usually associated with gastroenteritis; and species B (HAdV-B3 and HAdV-B7), C (HAdV-C1, HAdV-C2, and HAdV-C5), and E (HAdV-E4) are usually associated with respiratory diseases [2]. Recombination between members of the same species and between members of different species has been frequently described [4]. As a result, certain new types may acquire different pathogenicity and have strong potential for widespread and epidemic outbreaks. Consequently, surveillance of HAdV circulation with an early evaluation of the relationships between clinical manifestations and molecular characteristics of new infecting strains may be important for the development of adequate diagnostic, prophylactic, and therapeutic measures against HAdV infection.

HAdVs play an important role in the determination of respiratory infections, particularly in children. HAdVs are responsible for a number of lower respiratory tract diseases in children, including community-acquired pneumonia (CAP). Wo et al. reported that among 3,089 nasopharyngeal aspirates collected in children with CAP in China, 186 (6.0%) tested positive for HAdV [5]. Although HAdVs are associated with mild to moderate disease in most cases, life-threatening disease can occur in some patients, particularly if they are immunocompromised, [2]. Overall, little data on HAdV circulation have been collected in Europe and no recent data regarding the epidemiology, molecular characterization, and clinical features of respiratory HAdV infections in children have been collected in Italy. The main aim of this study was to evaluate the predominant HAdV species and types associated with pediatric respiratory infections in Milan, Italy, during two consecutive winter seasons. Clinical features related to HAdV types and genetic characteristics were also studied.

## Methods

### Study design

To evaluate the circulation of the different HAdV types and the possible relationship between viral load, viral genetic characteristics, and the severity of infection, nasopharyngeal swabs were collected from otherwise healthy children consecutively attending the Emergency Room of the Fondazione IRCCS Ca’ Granda Ospedale Maggiore Policlinico, University of Milan, Italy, due to a respiratory tract infection. The study was carried out during the period from January 1 to February 28 in two subsequent years, 2013 and 2014, and was approved by the Ethics Committee of the Fondazione IRCCS Ca’ Granda Ospedale Maggiore Policlinico, Milan, Italy. Written informed consent from a parent or legal guardian was required, and children ≥8 years of age were asked to give their written assent.

Patient demographic characteristics and medical histories were systematically recorded before the visit to the Emergency Room using standardized written questionnaires. The study patients were classified into disease groups (i.e., acute otitis media, rhinosinusitis, pharyngitis, croup, infectious wheezing, acute bronchitis, pneumonia) on the basis of signs and/or symptoms using well-established criteria and were subdivided into two subgroups: upper respiratory tract infections (URTIs) and lower respiratory tract infections (LRTIs) [6]. Nasopharyngeal secretions were collected from all of the children immediately after admission to the Emergency Room using a paranasal flocked swab (1 swab per child), which was stored in a tube containing 1 mL of universal transport medium (Kit Cat. No. 360c, Copan Italia, Brescia, Italy).

### Respiratory virus identification

Viral nucleic acids were extracted from the swab by means of a Nuclisens EasyMAG automated extraction system (Biomeriéux, Craponne, France), and the extract was tested for respiratory viruses using the respiratory virus panel (xTAG RVP Fast v2) (Luminex Molecular Diagnostics, Inc., Toronto, Canada) fast assay in accordance with the manufacturer’s instructions (Luminex Molecular Diagnostics Inc.) This assay simultaneously detects the following viruses: influenza A virus (FluA subtypes H1 or H3); influenza B virus (FluB); respiratory syncytial virus (RSV); parainfluenzaviruses (HPiV) 1–4; adenoviruses (HAdV); human metapneumovirus (hMPV); coronaviruses (hCoV) -229E, -NL63, -OC43, and -HKU1; enterovirus/rhinovirus (HEV/HRV) and human bocavirus (hBoV). Moreover, considering the risk of false negative results reported for the RVP Fast v2 Assay [7], the negative samples were also tested with an alternative HAdV-specific real time-PCR [8]. Positive results were also quantified with a HAdV-specific real-time polymerase chain reaction (PCR), as previously described [9].

Viral nucleic acid extracts were tested using a specific HAdV plasmid by a single-plex real-time PCR using TaqMan Universal Master Mix II (Applied Biosystems, Foster City, California, USA). Amplification and detection of viral DNA was performed with a 7900HT real-time PCR system instrument (Applied Biosystems). The real-time HAdV-specific primer sequences were as follows: 5’-GCCACGGTGGGGTTTCTAAACTT-3’, Adenoquant 1 (AQ1) and 5’-GCCCCAGTGGTCTTACATGCACATC-3’, Adenoquant 2 (AQ2). The sequence of the probe was 5’-TGCACCAGACCCGGGCTCAGGTACTCCGA-3’ (Adenoprobe) labeled with FAM on the 5’-end as a fluorescent dye and labeled with TAMRA on the 3’-end as a fluorescence quencher dye. Cycling conditions were as follows: 50°C for 2 min, 95°C for 8 min and 50 cycles of 95°C for 15 sec and 59°C for 1 min. The plasmid amplified target fragment was verified by sequencing [7]. Plasmid DNA concentrations were detected using an ND-1000 spectrophotometer (NanoDrop products, Wilmington, DE).

Real-time fluorescence quantitative PCR was carried out in a total reaction volume of 20 μL consisting of 10 μL of TaqMan Universal Master Mix (Applied Biosystems), 0.8 μL (0.4 mM) of each primer, 0.6 μL (0.3 mM) of the probe, 5 μL of template, and 2.8 μL of double-distilled water. The real-time PCR thermal cycling reaction and quantitative measurement were performed in a StepOne real-time PCR instrument (Applied Biosystems) using the following conditions: one cycle at 50°C for 2 min, one cycle at 95°C for 10 min, 45 cycles at 95°C for 15 s, and one cycle at 60°C for 1 min [9]. Each run included plasmid and negative controls. Standard precautions were taken throughout the PCR process to avoid cross-contamination. Negative controls and serial dilutions of the plasmid positive control were included in every PCR assay. Finally, quantitative results were reported as DNA copies/mL of respiratory samples.

### HAdV sequencing

HAdV typing was performed by sequencing the hypervariable region (1–6) of loop 1 of the hexon protein using a protocol proposed by Lu and Erdman [10]. PCR products ranging in size from 764 to 896 bp (first PCR) and 688 to 821 bp (nested PCR). First-round amplification was carried out using primers for AdhexF1 (nt 19135–19160; 5’-TICTTTGACATICGIGGIGTICTIGA-3’) and AdhexR1 (nt 20009–20030; 5’-CTGTCIACIGCCTGRTTCCACA-3’) followed by a nested round of PCR performed using internal primers: AdhexF2 (nt 19165–19187; 5’-GGYCCYAGYTTYAARCCCTAYTC-3’) and AdhexR2 (nt 19960–19985; 5’-GGTTCTGTCICCCAGAGARTCIAGCA-3’). PCR amplification was performed in a 50 μL reaction containing 45 μL of reaction mixture (10 mM Tris-HCl [pH 8.3], 1.5 mM of MgCl2, 50 mM KCl, 200 μM of each deoxynucleotide triphosphate, 0.2 μM of each primer, 1 U of Taq DNA polymerase [Roche Diagnostics, Indionapolis, IN]) and 5 μL of nucleic acid extract on a GeneAmp PCR System 9700 (Applied Biosystems) with the following settings: 94°C for 2 min denaturation followed by 35 cycles of 94°C for 1 min, 45°C for 1 min, and 72°C for 2 min, with a final extension of 72°C for 5 min. For the nested reaction, 0.5 μL of the first PCR product was amplified as above. Amplified products were separated on 1% agarose gels and purified with the QIAquick PCR Purification Kit (Qiagen, Chatsworth, California, USA). Sequencing was performed in both directions using AdhexF2/AdhexR2 primers and the ABI Prism BigdyeTM Terminator Cycle Sequencing Ready Reaction Kit Ver. 3.1 on an ABI 3100 DNA Sequencer (Applied Biosystems).

Sequencher 3.1.1 software (Gene Codes, Ann Arbor, Minnesota, USA) was used for sequence assembly and editing. All sequences were aligned using ClustalX 2.1 and BioEdit (version 7.1.3.0) software (Ibis Biosciences, Carlsbad, California, USA). Phylogenetic trees were generated using the Maximum likelihood method with Molecular Evolutionary Genetics Analyses (MEGA) software, version 5.05 [11], and Adenovirus prototype strains. Bootstrap probabilities for 1,000 iterations were calculated to evaluate confidence estimates. The graphs were made using GraphPad Prism version 5.01 for Windows (GraphPAD Software, San Diego, California, USA). All the HAdV sequences originated from this study were submitted to GenBank (accession numbers KT963953-KT964000).

### Statistical analysis

Descriptive statistics of the responses were generated. Continuous variables were presented as the mean values and standard deviations (SDs), and categorical variables were presented as numbers and percentages. For categorical data, comparisons between groups were performed using a contingency table analysis with a χ^2^ or Fisher’s exact test when appropriate. For ordered categorical data, a Cochran-Armitage test for trends was used to compare the groups. Continuous data were analyzed using a two-sided Student’s t-test after ensuring the data were normally distributed (based on the Shapiro-Wilk statistic) or using a two-sided Wilcoxon’s rank-sum test if the data were non-normal. All analyses were two tailed, and p-values of 0.05 or less were considered to be statistically significant. All analyses were conducted using SAS version 9.2 (Cary, NC, USA).

## Results

### HAdV incidence and phylogenetic analyses

During the two study periods, a total of 307 nasopharyngeal samples were collected in the emergency room. Of these, 61 (19.9%) tested positive for HAdV. The Luminex xTAG RVP Fast v2 assay identified 30 cases, all confirmed by real-time PCR. This method revealed 31 positive cases that tested negative with the RVP Fast V2 assay. Among the HAdV infected children, 14.8% were <1 year old, whereas 42.6% and 42.6% were 1–2 and ≥3 years old, respectively (Table 1).

**Table 1:** Number of children tested positive for human adenovirus (HAdV) with respiratory tract infection across different age groups, according to species and viral load.

	No. (%) of positive samples					
Age group (years)	Overall	Species B	Species C	Other species	Low viral load	High viral load
< 1	9 (14.8)	2 (28.6)	7 (18.9)	0 (0.0)	5 (13.5)	4 (18.2)
1–2	26 (42.6)	2 (28.6)	17 (45.9)	2 (50.0)	14 (37.8)	10 (45.4)
≥ 3	26 (42.6)	3 (42.8)	13 (35.2)	2 (50.0)	18 (48.7)	8 (36.4)
**Total**	61	7	37	4	37	22

Serotype was not available for 13 positive subjects, and viral load was not available for 2 positive subjects. Viral load was categorized in two groups and was considered “low” for values <6 log(copies/mL) and “high” for values ≥6 log(copies/mL). No statistically significant result emerged for the relationship between adenovirus types and age or between viral load and age.

The prevalence of HAdV detection was similar in the two studied periods: 28 (45.9%) and 33 (54.1%) positive samples were collected in 2013 and 2014, respectively. HAdV was the only virus detected in 31/61 (50.8%) cases, whereas it was found in association with one other virus in 25 (41.0%) cases and with two or more viruses in 5 (8.2%) cases. HEV/HRV and RSV were the most common co-infecting viral agents and were found in 12 (19.7%) and 7 (11.5%) samples, respectively.

**Fig 1:**
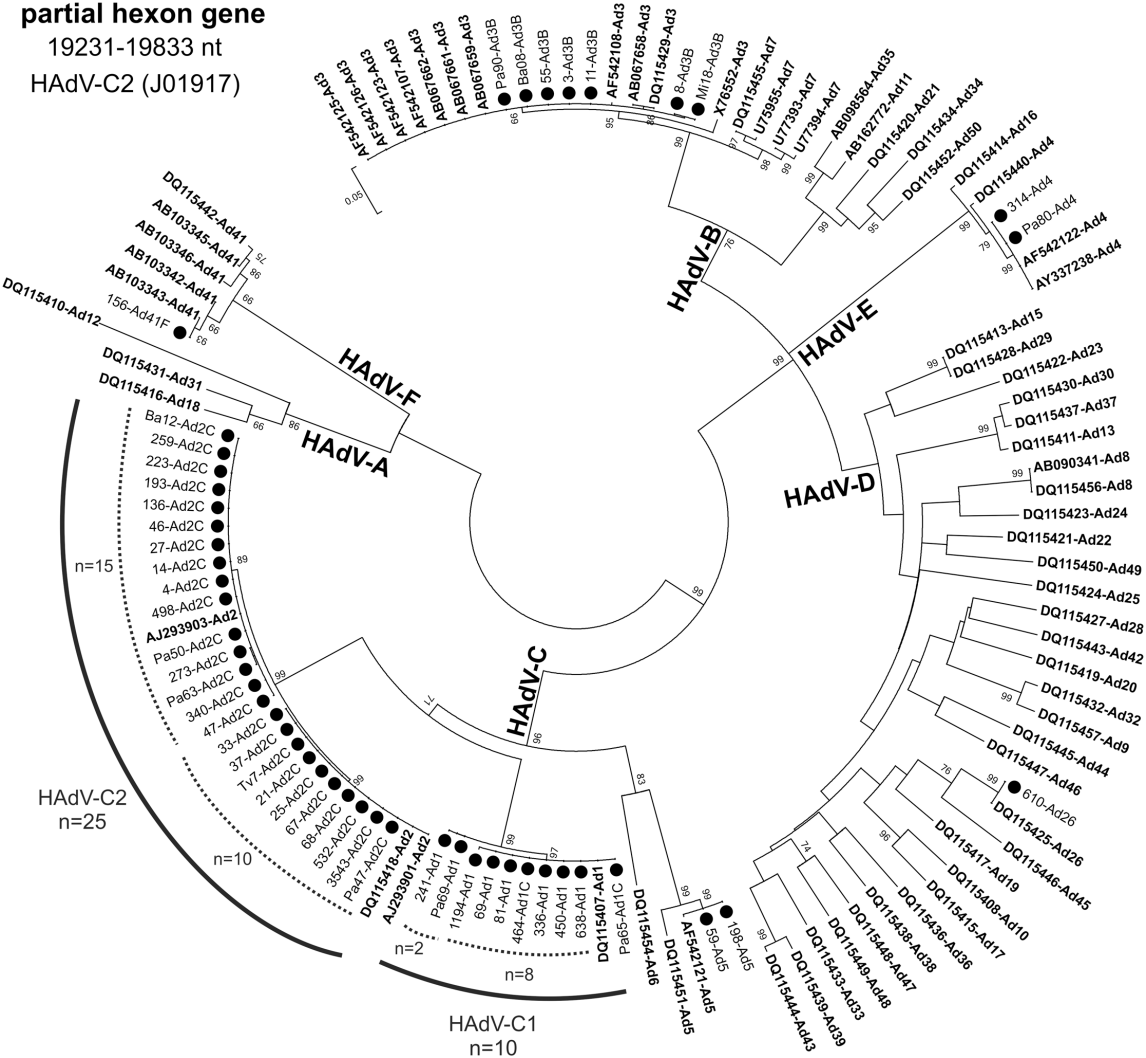
Phylogenetic tree based on partial hexon gene sequences of HAdV strains. Sequences originated from this study are indicated with black circles. HAdV reference stains are in bold. The percentage of replicate trees in which the associated taxa clustered together in the bootstrap test (1000 replicates) is shown next to the branches.

Molecular typing assignments were based on the identity of the closest matching sequences after both BLAST and phylogenetic analysis. The 61 HAdVs belonged to species B in 7 cases (11.5%; all HAdV-B3), species C in 37 cases (60.7%; 10 HAdV-C1, 25 HAdV-C2, and 2 HAdV-C5), species D in 1 case (1.7%; HAdV-D26), species E in 2 cases (3.4%; HAdV-E4), and species F in 1 case (1.6%; HAdV-F41) (Fig 1). It was not possible to identify the species and type of 13 (18.6%) samples due to inadequate sample volume.

No peculiar clustering was observed among HAdV strains. HAdVs circulating in 2013 were closely related to strains identified in 2014. However, among HAdV-C2 sequences, two distinctive branches were observed with 15 and 10 Italian strains (Fig 1). Similarly, in the branch of the tree corresponding to the HAdV-C1 strains, two strains appeared to cluster separately from the other 8 strains.

### Viral load

Using 10^6^ DNA copies/mL as a cut-off, the viral load was classified as low in 37 (62.7%) and as high in 22 (37.3%) cases (2 HAdV-B, 14 HAdV-C, and 7 of 11 without species identification). For HAdV-B, viral load varied from 1.4 x 10^2^ to 3.9 x 10^8^ copies/mL, whereas for HAdV-C it was between 3.4 x 10^3^ and 3.0 x 10^9^ copies/mL. No significant difference in viral load was observed between each HAdV species

The sequence identity matrix of the HAdV partial hexon gene for groups with at least 7 sequences (HAdV-C2, -C1 and -B3) showed a minimum to maximum identity range of 97.6–100.0% for HAdV-C2, 98.7–100% for HAdV-C1, and 99.4–100% for HAdV-B3.

Overall, the HAdV strain sequences analyzed were highly conserved and only few amino acid changes were observed. In detail, among the HAdV-C2 sequences, 15/25 strains (60.0%) had an insertion of one glutamic acid (E) in position 151 and the M305L change. In two of these strains, the additional change S195T was identified. Among the HAdV-C1 sequences, only one amino acid change (A190T) was evidenced in 2/10 strains (20.0%). Finally, in 5/7 strains (71.4%) belonging to the HAdV-B3 group, two changes, G205V and T254I, were observed.

### Clinical characteristics of HAdV infection

In Table 2, demographic, clinical, and laboratory characteristics of children infected by HAdV alone or co-infected with HAdV and one or more other respiratory viruses are compared with those of children with respiratory infection due to other agents. A preliminary analysis revealed that no statistically significant difference between cases infected by HAdV alone or co-infected with other viruses, in particular RSV or Rhinovirus could be evidenced, all the children with HAdV infection were considered together. As shown, in comparison to HAdV-negative children, those infected with HAdV were younger (4.3 ± 3.3 vs 3.2 ± 2.5 years; p = 0.01) and had high-grade fever more frequently (56.4% vs 72.4%; p = 0.03). Moreover, children infected with HAdV had lower respiratory tract involvement less frequently (55.2% vs 36.1%; p = 0.007) and never suffered from wheezing unlike children with disease due to other etiologic agents. Children infected with other agents wheezed in 12.5% of the cases (p = 0.004) and were hospitalized more frequently (56.1% vs 27.9%; p<0.001). No other significant differences between groups were observed.

**Table 2:** Comparison between subjects who tested positive and those who tested negative for human adenovirus (HAdV), according to demographic, clinical, and laboratory variables^a^.

Characteristic	HAdV-negative N = 248	HAdV-positive N = 61	P value for comparison
	n/N (%)	n/N (%)	
**Demographic and clinical presentation**			
Males (%)	143/244 (58.6)	29/60 (48.3)	0.15
Mean age ± SD, yrs	4.3 ± 3.3	3.2 ± 2.5	**0.01**
Presence of fever” (%)	211/220 (95.9)	56/58 (96.6)	0.99
High-grade fever° (%)	124/220 (56.4)	42/58 (72.4)	**0.03**
Respiratory rate, breaths/min	37.0 ± 11.7	34.7 ± 9.9	0.41
SpO_2_ in room air, mean % ± SD	97.2 ± 2.4	97.8 ± 1.9	0.17
Clinical findings			
Cough	180/236 (76.3)	43/60 (71.7)	0.46
Rhonchi	25/239 (10.5)	2/61 (3.3)	0.08
Rales	108/240 (45.0)	21/61 (34.4)	0.14
Wheezes	30/240 (12.5)	0/61 (0.0)	**0.004**
Diagnosis			
Upper respiratory tract infection	111/248 (44.8)	39/61 (63.9)	
Lower respiratory tract infection	137/248 (55.2)	22/61 (36.1)	**0.007**
**Clinical outcome**			
Hospitalization rate, no.(%)	139/248 (56.1)	17/61 (27.9)	**<0.001**
Drug use, no. (%)			
Antibiotics	225/248 (90.7)	53/61 (86.9)	0.37
Antipyretics	101/111 (91.0)	43/46 (93.5)	0.76
Aerosol therapy	48/111 (43.2)	14/46 (30.4)	0.14
Absence from child care attendance	6.1 ± 3.3	9.1 ± 7.0	0.07
mean days ± SD			
Similar illness within the households	45/111 (40.5)	16/48 (33.3)	0.39
**Laboratory data**			
White blood cell count (cells/μL)	14,589 ± 8,255	15,381 ± 7,461	0.40
Neutrophils, %	63.4 ± 19.9	60.3 ± 17.0	0.27
CRP, μg/dL	12.2 ± 25.8	23.3 ± 53.9	0.40

CRP, C reactive protein; SD, standard deviation; SpO_2_, peripheral oxygen saturation.”38.0°C or more any time during the illness (before or at enrolment, or during follow-up); °39.0°C or more any time during the illness (before or at enrollment or during follow-up).

^a^ Data were extracted from datasets of different studies that collected different information; therefore, the denominators vary across characteristics. However, when not indicated the reported number refers to the whole enrolled sample.

In Table 3, comparisons based on demographic, clinical, and laboratory variables between subjects with HAdV B and C species are shown. Two significant differences were found between the groups: antibiotic therapy was more frequently prescribed (91.9% vs 57.1%; p = 0.04) and white blood cell count was higher (17,244 ± 7,737 vs 9,565 ± 3,211; p = 0.04) in children infected by HAdV-C.

**Table 3:** Comparison between subjects with human adenovirus (HAdV) species B and C, according to demographic, clinical, and laboratory variables^a^.

Characteristic	HAdV-B species N = 7	HAdV-C species N = 37	P value for comparison
	n/N (%)	n/N (%)	
**Demographic and clinical presentation**			
Males (%)	4/7 (57.1)	19/36 (52.8)	0.99
Mean age ± SD, yrs	3.0 ± 1.9	2.8 ± 2.4	0.82
Presence of fever” (%)	5/5 (100.0)	34/36 (94.4)	0.99
High-grade fever° (%)	5/5 (100.0)	26/36 (72.2)	0.31
Respiratory rate, breaths/min	39.2 ± 8.5	34.9 ± 10.7	0.23
SpO_2_ in room air, mean % ± SD	96.6 ± 3.7	97.9 ± 1.8	0.61
Clinical findings			
Cough	4/7 (57.1)	25/37 (67.6)	0.67
Rhonchi	0/7 (0.0)	0/37 (0.0)	-
Rales	3/7 (42.9)	11/37 (29.7)	0.66
Wheezes	0/7 (0.0)	0/37 (0.0)	-
Diagnosis			
Upper respiratory tract infection	3/7 (42.9)	26/37 (70.3)	
Lower respiratory tract infection	4/7 (57.1)	11/37 (29.7)	0.21
**Clinical outcome**			
Hospitalization rate, no.(%)	3/7 (42.9)	9/37 (24.3)	0.37
Drug use, no. (%)			
Antibiotics	4/7 (57.1)	34/37 (91.9)	**0.04**
Antipyretics	3/4 (75.0)	27/29 (93.1)	0.33
Aerosol therapy	1/4 (25.0)	10/29 (34.5)	0.99
Absence from child care attendance, mean days ± SD	4.5 ± 0.7	9.6 ± 7.8	0.37
Similar illness within the households	1/5 (20.0)	13/30 (43.3)	0.63
**Laboratory data**			
White blood cell count (cells/μL)	9,565 ± 3,211	17,244 ±7,737	**0.04**
Neutrophils, %	57.6 ± 6.8	61.1 ± 17.8	0.99
CRP, μg/dL	5.9 ± 7.1	27.4 ± 69.7	0.87

CRP, C reactive protein; SD, standard deviation; SpO_2_, peripheral oxygen saturation.”38.0°C or more any time during the illness (before or at enrolment, or during follow-up); °39.0°C or more any time during the illness (before or at enrollment or during follow-up).

^a^Data were extracted from datasets of different studies that collected different information; therefore, the denominators vary across characteristics. However, when not indicated the reported number refers to the whole enrolled sample.

Table 4 shows data regarding characteristics of children with HAdV infection according to viral load. Children with high viral load were younger, had high-grade fever more frequently, were more frequently hospitalized, were absent from the community for a longer period of time, and had a higher C reactive protein (CRP) level. However, differences were statistically significant only for absence from the community (14.5 ± 7.5 vs 5.5 ± 3.2 days; p = 0.002) and CRP level (41.3 ± 78.5 vs 5.4 ± 9.6 μg/dL; p = 0.03).

**Table 4:** Comparison between subjects with low and high viral load, according to demographic, clinical and laboratory variables^a^.

Characteristic	Low viral load, <6 log(copies/mL) N = 37	High viral load, ≥6 log(copies/mL) N = 22	P value for comparison
	n/N (%)	n/N (%)	
**Demographic and clinical presentation**			
Males (%)	18/36 (50.0)	11/22 (50.0)	0.99
Mean age ± SD, yrs	3.4 ± 2.3	2.8 ± 2.7	0.16
Presence of fever” (%)	34/36 (94.4)	20/20 (100.0)	0.53
High-grade fever° (%)	25/36 (69.4)	16/20 (80.0)	0.39
Respiratory rate, breaths/min	34.6 ± 9.6	35.0 ± 11.1	0.99
SpO_2_ in room air, mean % ± SD	97.9 ± 1.4	97.5 ± 2.8	0.88
Clinical findings			
Cough	26/37 (70.3)	15/21 (71.4)	0.93
Rhonchi	1/37 (2.7)	0/22 (0.0)	0.99
Rales	10/37 (27.0)	10/22 (45.4)	0.15
Wheezes	0/37 (0.0)	0/22 (0.0)	-
Diagnosis			
Upper respiratory tract infection	27/37 (73.0)	12/22 (54.5)	
Lower respiratory tract infection	10/37 (27.0)	10/22 (45.4)	0.15
**Clinical outcome**			
Hospitalization rate, no.(%)	8/37 (21.6)	8/22 (36.4)	0.22
Drug use, no. (%)			
Antibiotics	31/37 (83.8)	20/22 (90.9)	0.70
Antipyretics	27/29 (93.1)	14/15 (93.3)	0.99
Aerosol therapy	7/29 (24.1)	7/15 (46.7)	0.18
Absence from child care attendance, mean days ± SD	5.5 ± 3.2	14.5 ± 7.5	**0.002**
Similar illness within the family	10/29 (34.5)	5/17 (29.4)	0.72
**Laboratory data**			
White blood cell count (cells/μL)	13,575 ± 7,624	15,891 ± 6,888	0.49
Neutrophils, %	62.6 ± 20.4	56.7 ± 13.5	0.38
CRP, μg/dL	5.4 ± 9.6	41.3 ± 78.5	**0.03**

CRP, C reactive protein; SD, standard deviation; SpO_2_, peripheral oxygen saturation.”38.0°C or more any time during the illness (before or at enrollment or during follow-up); °39.0°C or more any time during the illness (before or at enrollment or during follow-up).

^a^Data were extracted from datasets of different studies that collected different information; therefore, the denominators vary across characteristics. However, when not indicated the reported number refers to the whole enrolled sample

## Discussion

Several previous epidemiological studies have shown that HAdVs are considered the cause of respiratory infections in otherwise healthy children in approximately 4–10% of the cases [12–15]. In this study, similarly to what has been found in Asia by other authors [16], a prevalence of approximately 20% was found, suggesting that the relevance of this infectious agent in the determination of respiratory problems could be higher than previously thought. The methods used to identify HAdVs might partially explain this finding. In the past, most of the epidemiological studies of viral respiratory infections were carried out using methods that could underestimate viral presence in respiratory secretions, such as viral culture, antigen detection by immunofluorescence, and visualization by electron microscopy [17]. To overcome this problem, molecular methods were suggested. Multiplex assays, including the RVP Fast v2 assay, were developed to obtain the simultaneous identification of all the most common respiratory viruses and are now commonly used in routine practice. As previously reported [8] and confirmed by this study, the RVP Fast v2 assay has poor sensitivity for HAdV and can lead to undervaluation of the real importance of these viruses in the determination of respiratory infections. The addition of a specific real-time PCR can solve this issue.

Moreover, the higher than expected prevalence of HAdV infection evidenced by this study could be strictly related to the period during which it was carried out. Samples were collected in two winter months of two consecutive years. Although HAdVs circulate during the whole year, peak periods are in winter and early spring [1]. Consequently, it is possible that the study was carried out during epidemics leading to the higher prevalence values reported here.

In this study, the most commonly identified species were B and C, types 3, 2, and 1. This is not surprising because, despite possible temporal and regional changes in predominant type [18], these types are more commonly reported as the cause of respiratory infection worldwide. HAdV-B3 has been identified in successive outbreaks of severe acute respiratory illnesses in Korea [19, 20], Brazil [21], and Taiwan [22, 23], where this virus was the predominant type for respiratory HAdV infection from 1981 to 2002. Moreover, together with other HAdV types, it has been the causative agent in epidemic outbreaks of respiratory diseases in Europe, America, and Oceania [24–26]. Finally, HAdV-B3 is known to be a causative agent of a characteristic syndrome of acute pharyngo-conjunctival fever in older children and adults, especially in summer camps and swimming pools [27]. Types C1 and C2 have been more frequently reported as the cause of endemic or sporadic cases [28], although there have been reports of epidemics [19]. In Italy, a survey carried out approximately 10 years ago found that HAdV-C1 and -C2 were the most common HAdVs isolated in patients with infection due to these agents [28]. The same was shown by this study, showing that epidemics of infection due to the same HAdV in a given geographic area can be prolonged compared with outbreaks of RSV, parainfluenza virus, and influenza viruses, which are well defined and have duration limited to some months [29, 30].

The severity of HAdV infection varies according to age, socioeconomic status, environmental status, and above all, the immunological characteristics of the patient. Detection of HAdV in severely immunocompromised children has been implicated as a risk factor for poor outcome [31]. However, severe cases have been frequently described in otherwise healthy children [5, 19, 20]. In this study, most of the children with HAdV infection had a mild infection and, globally, the severity of respiratory infection of children in whom HAdV alone or in association with other respiratory viruses was identified was lower than that due to other infectious agents considered together. Both the prevalence of LRTIs and hospitalization rate were significantly lower in HAdV-infected children than in children not infected by HAdV. The long-term circulation of HAdVs with similar genetic characteristics could partially explain the generally poor clinical relevance of infections due to the strains identified in this study. In Italy, the most common HAdVs identified in children during the periods of this study were of the same species and of the same types of those identified several years before. Moreover, despite sequencing analyses that were focused on one of the more variable genes (hexon) [32], no significant variation in HAdV genetic characteristics was seen and no recombination between viruses was found. HAdV-C2 strains were the only strains to have two slightly different clusters, suggesting the circulation of two different HAdV-C2 strains with indistinct pathogenetic roles. As a result, it is possible that many of the children had previously had contact with these viruses and developed sufficiently high immunity to limit the clinical expression of subsequent infections. However, the number of non-HAdV infection children included in this study is significantly higher than that of HAdV infected patients and this difference could have led comparison to wrong results. A longer period and expanded surveillance may help to construct the complete picture on the HAdV circulation, other infections and related clinical features.

Prevalence of LRTI was higher in children infected by type B3, although the difference compared with type C was not statistically significant and the total number of patients with this type of infection is too small to draw definitive conclusions. Potentially increased virulence of type B3 in comparison to other HAdVs is not surprising because this virus has already been associated with a number of severe LRTIs [5, 19, 20] and to the development of acute meningo-encephalopathy [33].

In this study, higher viral load was more common in children with some markers of more severe disease, such as higher fever, higher hospitalization rate, higher CRP values, and delayed return to normal activities, independent of the infecting HAdV type. However, clinical differences between patients with high or low viral load were not always significant, and it is not possible to state that HAdV load can be a marker for severity of infection. By contrast, HAdV load was found to be significantly higher in patients developing severe HAdV infection after transplantation, especially in pediatric stem cell transplant recipients [31]. Consequently, the measurement of HAdV load is considered a possible method for an early diagnosis of disseminated HAdV disease and for the initiation and monitoring of antiviral therapy in these subjects [34]. Further studies are needed to evaluate whether high viral load could indicate which subjects might have to receive antiviral therapy to avoid negative evolution of the infection even if they are not immunocompromised.

In conclusion, this study has shown that when adequately investigated, HAdV infections are diagnosed more commonly than usually thought. Moreover, these data seem to indicate that HAdVs are stable infectious agents that do not frequently incur genetic variations and, for this reason, do not cause frequently severe diseases. It seems that there are some differences in the severity of disease outcome between types and according to viral load, with HAdV type C and high viral load apparently associated with a more severe disease. However, further studies are needed to identify the potential pathogenetic role of the different species and types of HAdV and the importance of viral load in the severity of infection. Clarification of these unsolved problems may be useful for deciding how to develop adequate preventive and therapeutic measures for immunocompromised and otherwise healthy children who suffer from HAdV infection.
